# Intracranial vein of Galen malformation and its management: A case report

**DOI:** 10.3892/mi.2024.187

**Published:** 2024-08-05

**Authors:** Zenia A. Elavia, Rohan Raj, Muhammad Ayaz Tariq, Moyal Zehra Saad, Preeti Kumari Yadav, Jubran Al Hooti, Hend Makky

**Affiliations:** 1 Department of Internal Medicine, Dr. DY Patil Medical College, Pune, Maharashtra 411018, India; 2 Department of Internal Medicine, Nalanda Medical College and Hospital, Patna, Bihar 800001, India; 3 Quaid-e-Azam Medical University, Bahawalpur, Punjab 63100, Pakistan; 4 Department of Internal Medicine, Jinnah Medical and Dental College, Karachi, Sindh 75510, Pakistan; 5 Department of Internal Medicine, Cama and Albless Hospital, Mumbai 400001, India; 6 School of Medicine, University College Dublin, Dublin D04 V1W8, Ireland; 7 Faculty of Medicine, Assiut University, Assiut 71515, Egypt

**Keywords:** arteriovenous malformation, endovascular embolization, hydrocephalus, vein of Galen, ventriculoperitoneal shunt

## Abstract

Vein of Galen malformation (VOGM) is a rare congenital arteriovenous malformation affecting the pediatric population, characterized by a fistula between the diencephalon and a dilated vein of Galen. The present study reports the case of a 6-month-old male infant referred for developmental delays and abnormal head circumference. A clinical examination revealed macrocephaly, dilated scalp veins and neurological abnormalities. Neuroimaging confirmed a large VOGM with associated hydrocephalus. A multidisciplinary team devised a treatment plan involving endovascular embolization and ventriculoperitoneal shunt placement. The post-operative recovery exhibited an improvement in neurodevelopmental milestones and a reduced head circumference. Generally, the management of VOGM requires a comprehensive approach, including early diagnosis, precise imaging and timely intervention. The case described herein highlights the importance of interventional radiology in planning a management plan and the novel kissing microcatheter endovascular embolization technique.

## Introduction

The vein of Galen is an intracerebral vein that drains blood from the cerebral hemispheres and the basal ganglia (1). It is formed by the fusion of the internal cerebral veins and the basal veins of Rosenthal. Vein of Galen malformation (VOGM) is an arteriovenous malformation with a direct connection between the diencephalon and a dilated vein of Galen (1). Steinheil first discovered VOGM in 1895, accounting for 1% of pediatric congenital malformations (2).

VOGMs occur when the prosencephalic vein of Markowski, the fetal precursor of the vein of Galen, is maldeveloped (3). At ~3 months of gestation, the prosenchephalic vein of Markowski regresses and forms the vein of Galen. However, in cases where there is an arteriovenous malformation, the vein of Galen receives high blood pressure, which it is not well adapted for, and, as a result, dilates (3). This leads to increased venous blood pressure and results in a whole array of symptoms, most commonly increased cerebral pressure. Delayed neurodevelopment and even the loss of brain tissue can result from VOGM. Another common manifestation is heart failure (4). This is due to the shortened circuit of venous return to the heart and the increased venous pressure, all of which increase the preload on the neonatal heart, causing high-output heart failure within the first few days of life (5).

Currently, embolization is the main treatment method for VOGM. Various studies have been conducted on embolization techniques, such as transarterial and transvenous (6). Usually, there is a follow-up period of 6 months post-treatment. However, the rate of complications remains high, with almost 41% of patients suffering from complications even 15 years following treatment (7). The present study describes the case of a 6-month-old male infant with VOGM and discusses the management techniques of VOGM and evaluates prognosis.

## Case report

### Patient history

A 6-month-old male African American infant was referred to the Department of Neurology, Nalanda Medical College (Patna, India) clinic due to concerns regarding developmental delays and an abnormal head circumference. The parents of the child reported a progressive increase in head size since birth. Further investigation revealed a systolic murmur and prominent scalp veins.

### Clinical examination

A physical examination confirmed macrocephaly, bulging fontanelles and dilated scalp veins. A bruit was auscultated over the anterior fontanelle, suggesting a vascular abnormality. A neurological examination revealed hypotonia and delayed developmental milestones.

### Diagnostic workup

Neuroimaging was conducted to locate the suspected vascular abnormality, and an immediate assessment was conducted with a computed tomography (CT) scan to confirm its anatomical position. The CT ruled out calcifications and bone structure abnormalities, and helped to assess whether any complications had already arisen, such as extensive hydrocephalus or a hemorrhage. The patient underwent magnetic resonance imaging (MRI) with contrast, which revealed a large arteriovenous malformation involving the vein of Galen, causing the dilatation of the deep venous system and hydrocephalus. The MRI provided high-resolution images, particularly T1 and T2 weighted, and displayed soft tissue contrast, which helped precisely distinguish the malformation from surrounding structures. Subsequently, a magnetic resonance angiography (MRA) was conducted to accurately map out the angioarchitecture, including the feeding arteries, draining arteries and the size of the vein of Galen aneurysm. This displayed multiple abnormal arterial connections feeding into the dilated vein of Galen, which when considering the presenting features of hemodynamic impact on the heart and macrocephaly, is suggestive of choroidal type of VOGM.

**Figure 1:**
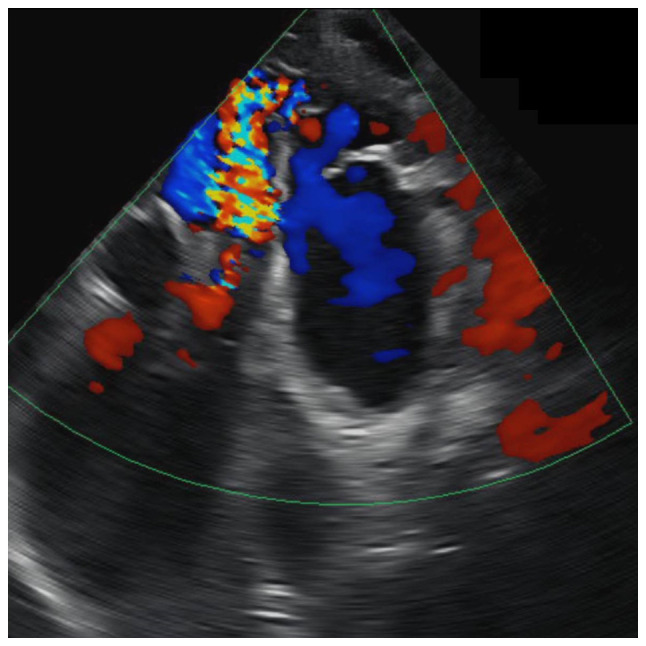
Transthoracic echocardiograph of the patient.

Hemodynamics were assessed, and a treatment plan, the endovascular embolization, was devised based on the vascular mapping. Finally, a brain arteriovenous malformation angiogram was carried out to further visualize the vascular network and identify the feeding and draining arteries. This is the gold standard for planning endovascular embolization. An additional echocardiography was conducted (Fig. 1), to assess the extent of heart involvement and whether the heart failure was manageable. No typical heart failure was observed; however, there was a high-flow cardiac murmur, which confirmed the hemodynamic impact of the arteriovenous malformation.

### Neuroimaging

**Figure 2:**
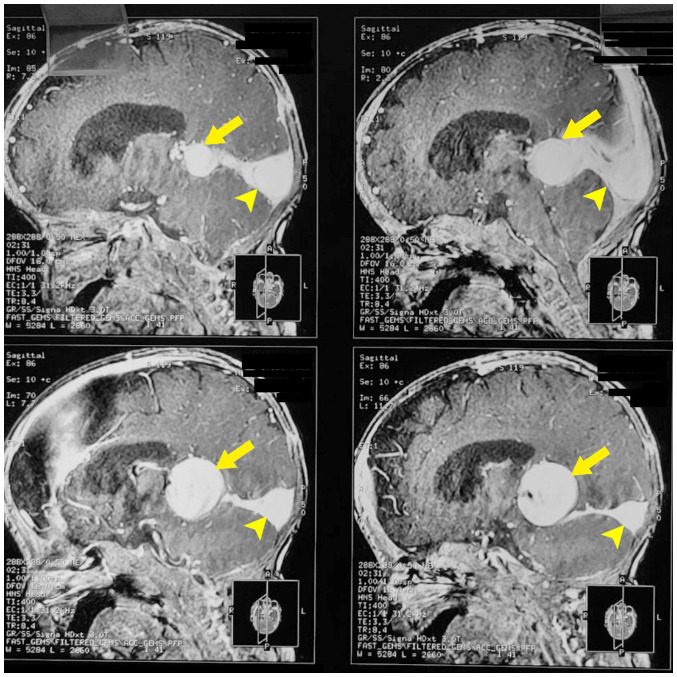
Sagittal T2-weighted magnetic resonance imaging. The dilated galenic vein (yellow arrow), also known as the median vein of prosencephalon, is located midline in the cistern of the velum interpositum and drains into the superior sagittal sinus (yellow arrowhead).

The axial CT scan displayed hyperdense appearances within the vascular channels, which are the abnormally dilated venous structures in cerebral arteriovenous malformation, the VOGM. MRI scans carried out on the patient demonstrated a markedly dilated median prosencephalic vein. The sagittal MRI T2-weighted images are presented in Fig. 2. The dilated galenic vein (yellow arrow; Fig. 2), also known as the median vein of prosencephalon, is located midline in the cistern of the velum interpositum and drains into the superior sagittal sinus (yellow arrowhead; Fig. 2).

An MRA was performed (image not available) and this revealed multiple enlarged arterial branches from the anterior and posterior cerebral arteries coalescing on the lateral margins of the dilated VOGM.

**Figure 3:**
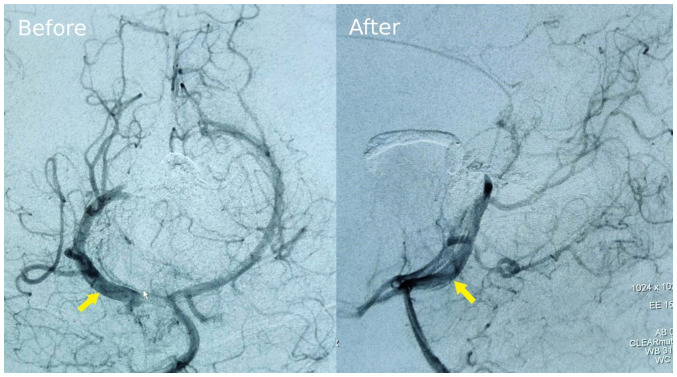
Brain arteriovenous malformation angiogram at 6 months of age and 2 weeks after endovascular embolization. The yellow arrow indicates the vein of Galen malformation.

Of note, two brain artery malformation angiograms were performed (Fig. 3). The image on the left panel in Fig. 3, taken at 6 months of age, displays the extensive network of abnormal arterial connections feeding into the dilated median prosencephalic vein. This is suggestive of the choroidal type of VOGM rather than mural. The image on the right panel in Fig. 3, taken 2 weeks post-intervention, demonstrates a reduction in the size and complexity of the malformation, indicating successful embolization of the feeding vessels. The yellow arrow points to the VOGM.

### Diagnosis

The patient was diagnosed with choroidal VOGM with associated hydrocephalus.

### Management

A multidisciplinary team consisting of neurosurgeons, interventional neuroradiologists and pediatric cardiologists assessed the case and evaluated possible treatment plans. Currently, the gold-standard treatment for VOGM is endovascular embolization, and since the patient was 6 months old, which is the optimum age for carrying out the procedure, the decision was finalized. A surgical strategy was devised with the aid of the MRA and arteriovenous malformation angiogram. An endovascular embolization was performed, using a combination of transarterial and transvenous routes, known as the kissing microcatheter technique, to occlude abnormal vessels feeding into the malformation, reducing blood flow and improving symptoms. In addition, a ventriculoperitoneal shunt was placed to alleviate hydrocephalus and control intracranial pressure. Following the procedure, another arteriovenous malformation angiogram was conducted to assess the situation and determine the success of the intervention. Finally, regular follow-up appointments and imaging studies were scheduled every 2 months for the subsequent 6 months to monitor the patient's neurological development and assess the effectiveness of the intervention.

### Outcome

The post-operative recovery was marked by an improvement in the neurodevelopmental milestones and a reduction in the head circumference of the child. Follow-up imaging demonstrated the successful embolization of the malformation with decreased arteriovenous shunting. The bi-monthly follow-up appointments demonstrated that the patient tolerated the procedure well and is showing significant clinical improvement in his appetite, speech, and cognitive functions. He did not experience any seizure activity, and his follow-up echocardiology revealed an improvement with no residual high-flow murmur.

## Discussion

VOGM presents with various manifestations, all of which are detrimental to fetal growth and neurodevelopment. Although the onset of pathophysiology is *in utero*, numerous signs and symptoms arise after birth when the protective low-resistance circulation generated by the placenta is removed (8). One of the more significant manifestations is high-output cardiac failure (5). This is a result of the increased venous pressure and venous return to the heart, causing right-sided heart failure. The increased preload to the heart can display different degrees of damage depending on the size of the fistula in the VOGM. Small shunts are associated with an improved prognosis and usually present at a later stage in the neonate's development, with tachycardia and cardiomegaly (4). Larger shunts are associated with a worse prognosis and present at a much earlier stage with heart failure. The main manifestation of VOGM is hydrocephalus, usually remedied by installing a ventriculoperitoneal shunt to alleviate the pressure (8). Galen's dilating vein in an aneurysmal manner leads to aqueduct compression and subsequent abnormal CSF flow and venous congestion. This manifests as seizures and developmental abnormalities, and in severe cases, it can lead to ‘melting brain’ syndrome (9). This results from venous hypertension, and blood flow is mainly directed to the fistula, causing ischemic damage and loss of brain tissue, commonly called ‘melting brain’ syndrome (10).

There are two main types of VOGM: The choroidal and mural types (11). The choroidal type is where multiple fistulas feed into the VOGM, and it is more severe as it has a higher risk of causing heart failure, as well as other symptoms, such as macrocephaly and dilated orbital veins (12). The case described herein was classified as choroidal VOGM, as multiple fistulas were feeding into the vein of Galen aneurysm and the patient presented with classical features of heart involvement and macrocephaly. By contrast, the mural type usually presents with one fistula in the wall of the vein of Galen and is less severe, as it restricts more blood flow, but at the cost of greater dilatation (13). In such cases, there is a lower risk of heart failure; however, such cases usually present with macrocephaly, hydrocephaly, seizures and commonly, with developmental delays.

An ultrasound is usually the first investigation carried out when suspecting fetal central nervous system abnormalities; however, it is not as sensitive as a fetal MRI. Currently, the classic presentation of VOGM would be found prenatally in the third trimester with a fetal MRI or CT scan if needed (14). This is more advantageous as it displays the anatomy of the brain and provides a clearer image of the damaged and abnormal structures (15). MRA/MRV angiography is the gold standard for gauging a better view of the angioarchitecture, which is necessary to plan the endovascular procedure to treat the malformation (16). Angiography plays a crucial role in the diagnosis and evaluation of the stage of the disease (17). Yuval *et al* (18) identified various prognostic features that help better predict the course of the disease, and the two most important are the number of feeding arteries and the volume of venous drainage. Of note, more than five arteries feeding into the VOGM can be considered an indirect indication of potential massive shunting, which will likely lead to severe congestive heart failure (CHF). Furthermore, the less obstructed the venous drainage, the greater the volume overload and returning blood pressure of the heart, which increases the risk of developing CHF postnatally. Fetal echocardiography is also the gold standard for gauging the extent of cardiological involvement, which could indicate the nature of the VOGM (14).

Endovascular embolization is currently the only well-established treatment for VOGM (8). Prior to the development of endovascular embolization, the mortality rate of patients suffering from VOGM was almost 100% (4). Since the introduction of endovascular embolization, the prognosis of patients with VOGM has markedly improved over time, with the mortality decreasing from 17 to 12% and post-embolization complications decreasing from 45 to 35% in the 1980s and 2000s, respectively (19). The main complications reported for post-embolization were hematomas (37%), cerebral ischemia (6%) and hydrocephalus (3%). Yan *et al* (19) reported that good clinical outcome percentages increased substantially from 49 to 70% across the same period of time. A previous systematic review of endovascular embolization performed for 667 patients with VOGM between 1987 and 2014 demonstrated that 23-70% of the neonates were neurologically normal (20). In an adjacent 15-year study, those who did not receive endovascular embolization demonstrated a poor prognosis (21). A recent study by Lasjaunias *et al* on 233 patients with VOGM receiving endovascular embolization reported 10.6% mortality, and 74% were neurologically normal. The complications were mainly delayed development and psychomotor impairments (10).

Endovascular embolization is carried out at ~6 months of age unless there is an emergency situation that would require earlier endovascular embolization, such as congestive heart failure, that is refractory to medication. The aim is to ensure there are no developmental delays caused by cerebral venous hypertension and that the heart failure is manageable and not terminal (8). The agents used to embolize the fistulas are N-butyl-cyanoacrylate or onyx (22). Another more recent agent used is detachable micro-coils; however, these are associated with a higher risk of rupture and longer procedure durations. There are two routes with which VOGM can be accessed for endovascular embolization: Transarterial and transvenous, both of which achieve heart failure control in different situations. The transarterial route is more suitable for a small number of arterial feeders in the VOGM, whilst the transvenous one is more suitable for VOGM with many small arterial pedicles feeding into the fistula (3). The transvenous route is less favorable, as it has the associated risk of impairing deep venous drainage and subsequent aneurysm perforation (8). Currently, a combination of transarterial and transvenous routes, known as the kissing microcatheter technique has demonstrated promising results (6). Near complete angiographically confirmed closure of the VOGM in 79% of patients, and 69% reported normal outcomes post combination endovascular embolization routes.

The patient described herein presented with classical features of VVOGM that align with previous case reports on VOGM, which was later confirmed with MRI scans and echocardiography. The post-operative recovery was marked by an improvement in the neurodevelopmental milestones and a reduction in the head circumference of the child. Follow-up imaging demonstrated the successful embolization of the malformation with decreased arteriovenous shunting. This is a novel case as the interventional radiology specialty approach demonstrates the optimum diagnostic and preplanning investigations for cases of vascular origin. The learning point to appreciate is the need for interdisciplinary collaboration, particularly between interventional radiologists and neurosurgeons. Furthermore, the benefit of conducting a comprehensive radiological study of the case to optimize planning for a complex surgery. In addition, the kissing microcatheter endovascular embolization technique that was performed highlights the importance of considering this innovative surgical option in complex vascular cases. The limitation of this approach is the duration of these procedures that, in emergency cases, would be overlooked for more definitive management rather than undertaking an investigative approach. However, it is important to note that in the majority of cases of VOGM, if hemodynamic impact is minimal and manageable, then waiting until the patient is 6 months old is the convention as it is the optimal age for surgical intervention. Therefore, there is sufficient time to carry out a comprehensive neuroimaging investigation.

A limitation of the present study was the lack of an ability to generalize, as this is a case report meant to demonstrate a case of optimal management of VOGM with specific focus on interventional radiology and the kissing catheter endovascular embolization technique. Furthermore, there is no possibility to establish better treatment efficacy, as cases with VOGM are critical, with no room for trialing, as the patients are at an age of critical neurological development.

In conclusion, intracranial VOGM poses a complex challenge requiring a multidisciplinary approach for optimal management. In conjunction with interventional radiology, endovascular embolization has proven to markedly improve outcomes in affected infants. The present case report demonstrates the novel and efficient kissing microcatheter endovascular embolization technique and that suggests that this is a surgical option that could be considered more often when devising management plans. Furthermore, long-term follow-up is crucial to monitor potential complications and ensure ongoing neurodevelopmental progress.

## Data Availability

The datasets used and/or analyzed during the current study are available from the corresponding author on reasonable request.
